# Regulatory T cell frequency in patients with melanoma with different disease stage and course, and modulating effects of high-dose interferon-α 2b treatment

**DOI:** 10.1186/1479-5876-8-76

**Published:** 2010-08-16

**Authors:** Paolo A Ascierto, Maria Napolitano, Egidio Celentano, Ester Simeone, Giusy Gentilcore, Antonio Daponte, Mariaelena Capone, Corrado Caracò, Rosa Calemma, Gerardo Beneduce, Margherita Cerrone, Vincenzo De Rosa, Giuseppe Palmieri, Giuseppe Castello, John M Kirkwood, Francesco M Marincola, Nicola Mozzillo

**Affiliations:** 1 Unit of Medical Oncology and Innovative Therapy and Melanoma Cooperative Group, National Tumor Institute, Naples, Italy; 2 Institute of Biomolecular Chemistry-CNR, Trav. La Crucca, 3 - Baldinca Li Punti, Sassari, Italy; 3 Department of Medicine, Division of Hematology/Oncology, University of Pittsburgh Cancer Institute, Pittsburgh, PA, USA; 4 Department of Transfusion Medicine, Clinical Center, National Institute of Health, Bethesda, MD, USA

## Abstract

**Background:**

High-dose interferon-alpha 2b (IFN-α 2b) is the only approved systemic therapy in the United States for the adjuvant treatment of melanoma. The study objective was to explore the immunomodulatory mechanism of action for IFN-α 2b by measuring serum regulatory T cell (Treg), serum transforming growth factor-β (TGF-β), interleukin (IL)-10, and autoantibody levels in patients with melanoma treated with the induction phase of the high-dose IFN-α 2b regimen.

**Methods:**

Patients with melanoma received IFN-α 2b administered intravenously (20 MU/m^2 ^each day from day 1 to day 5 for 4 consecutive weeks). Serum Treg levels were measured as whole lymphocytes in CD4^+ ^cells using flow cytometry while TGF-β, IL-10, and autoantibody levels were measured using enzyme-linked immunosorbent assays.

**Results:**

Twenty-two patients with melanoma received IFN-α 2b treatment and were evaluated for Treg levels. Before treatment, Treg levels were significantly higher in patients with melanoma when compared with data from 20 healthy subjects (*P *= 0.001; Mann-Whitney test). Although a trend for reduction of Treg levels following IFN-α 2b treatment was observed (average decrease 0.29% per week), statistical significance was not achieved. Subgroup analyses indicated higher baseline Treg levels for stage III versus IV disease (*P *= 0.082), early recurrence versus no recurrence (*P *= 0.017), deceased versus surviving patients (*P = *0.021), and preoperative neoadjuvant versus postoperative adjuvant treatment groups (not significant). No significant effects were observed on the levels of TGF-β, IL-10, and autoantibodies in patients with melanoma treated with IFN-α 2b.

**Conclusions:**

Patients with melanoma in this study showed increased basal levels of Treg that may be relevant to their disease and its progression. Treg levels shifted in patients with melanoma treated with IFN-α 2b, although no firm conclusions regarding the role of Tregs as a marker of treatment response or outcome can be made at present.

## Background

High-dose interferon (HDI)-alpha 2b (IFN-α 2b) is the only approved adjuvant systemic therapy for resected, high-risk melanoma in the United States [1]. The approved regimen for HDI consists of an induction phase of 20 MU/m^2 ^intravenously (iv) 5 times per week for 4 weeks, followed by a maintenance phase of 10 MU/m^2 ^subcutaneously (sc) 3 times per week for 48 weeks [1]. In some European countries (Germany, Austria, Switzerland, and France) the standard of care for the adjuvant treatment of melanoma tends to be low-dose IFN (LDI; 3 MU per day 3 times each week), while neither HDI nor LDI is approved for use in other countries (e.g., United Kingdom and The Netherlands) [2].

Efficacy data from several pivotal trials have shown that adjuvant IFN-α 2b [3,4] and pegylated interferon-α 2b (Peg-IFN-α 2b) [5] significantly prolong relapse-free survival (RFS), but not overall survival (OS) compared with observations in high-risk patients with melanoma. These findings were reinforced in 2 separate meta-analyses of randomized trials investigating IFN-α 2b versus observation in high-risk patients with melanoma [6,7]. Some studies with IFN have shown evidence for an OS benefit. For example, the E1684 trial of HDI (with IFN-α 2b) in high-risk patients with melanoma demonstrated a statistically significant RFS and OS benefit [8], and in a recent study in patients with melanoma that had spread to the regional lymph nodes, LDI (with IFN-α 2a) given sc 3 times a week for 2 years significantly improved OS and disease-free survival (DFS) [9].

An individual patient data meta-analysis of randomized melanoma trials, covering a wide range of IFN dose regimens, suggested that the benefits of IFN are independent of dose or therapy duration, and translate into an absolute OS benefit of approximately 3% (95% confidence interval [CI]: 1%-5%) at 5 years [10].

Optimal dose and duration of IFN-α 2b therapy are not yet clear [2,11,12], but a better understanding of the mechanism of action may help to potentiate the clinical efficacy and reduce the toxicity [13] of IFN-α 2b/Peg-IFN-α 2b. Numerous studies suggest that the mechanism of action of IFN in melanoma is primarily immunomodulatory [14-18]. Efforts to elucidate this mechanism of action have focused upon the modulation of signal transducers and activators of transcription signaling and immunoregulatory responses mediated by regulatory T cells (Tregs) [19,20].

Recent evidence for the possibility of IFN acting through an indirect immunomodulatory mechanism has been reported [17,18]. In the Hellenic Oncology Cooperative Group trial [17], the development of autoantibodies or clinical manifestations of autoimmunity were associated with statistically significant improvements in RFS and OS in the IFN-α 2b induction only treatment arm as well as in the extended IFN-α 2b arm. Additionally, Moschos *et al*. [18] demonstrated that clinical responders treated with neoadjuvant IFN-α 2b had significantly greater increases in endotumoral CD11c^+ ^and CD3^+ ^cells and significantly greater decreases in endotumoral CD83^+ ^cells compared with nonresponders. However, a recently published subanalysis of the European Organization for Research and Treatment of Cancer (EORTC) 18952 and Nordic IFN trials suggests that appearance of autoantibodies was not strongly associated with improved clinical outcome when a correction was made for guarantee-time bias [21]. These data are contrary to the findings of Gogas *et al*. [17]; it should be noted, however, that the data were obtained from subsets of patients using different assays performed in separate laboratories, whereas the Hellenic trial data were from a prospectively designed study, obtained on full patient sets without exclusions. Further evidence for the induction of autoimmunity by IFN-α 2b was observed in the Eastern Cooperative Oncology Group (ECOG)-intergroup E2696 phase II trial which suggested that autoimmunity was a predictive biomarker of RFS with HDI when compared with the GM2-KLH/QS-1 (GMK) vaccine [16].

Tregs are a suppressive CD4^+ ^T cell population that is present, along with primed effector T cells, in tumor and tumor-draining lymph nodes [22]. Tregs express high levels of surface antigens such as CD25, cytotoxic T lymphocyte-associated antigen 4 (CTLA-4), and glucocorticoid-induced tumor necrosis factor receptor (GITR) [23,24]. Tregs also express a characteristic intracellular nuclear transcription regulator, forkhead box p3 (Foxp3) [25,26]. The presence of Tregs in tumor-draining lymph nodes and tumors may serve as a basis of potential inhibition of host effector cell function. Thus, depletion of Tregs or blockade of Treg function using antibodies or other strategies targeting Tregs might abrogate this Treg suppression and enhance antitumor immunity [27]. A recent study published by Cesana *et al*. [28] demonstrated that melanoma and renal cell carcinoma (RCC) patients had increased basal Treg levels. There was also a reduction in Treg levels in those patients who achieved an objective clinical response to high-dose bolus interleukin (IL)-2 therapy [28]. Another study investigated the effects of IFN-α and IL-2 therapy on Treg levels in patients with RCC [29]. Patients who responded to IFN-α therapy had lower Treg cell levels before treatment than did patients whose disease progressed, these results suggest that low Treg levels before IFN-α treatment may be a prognostic factor for better clinical outcome in patients with RCC [29].

Previously, we reported preliminary data indicating a decrease in peripheral blood Treg levels in patients with melanoma treated with HDI [14,30]. In order to explore clinical outcome in relation to Treg levels, we conducted a translational study in patients with stage III or IV melanoma. We examined whether neoadjuvant (before surgery) or adjuvant (after surgery in patients with no evidence of disease) therapy with the iv induction phase of the U.S. Food and Drug Administration (FDA)-approved HDI regimen affected the number of Treg cells in the peripheral blood. As secondary analyses, the effects of IFN-α 2b on serum transforming growth factor-β (TGF-β), IL-10, and autoantibody levels were also measured, along with efficacy and safety.

## Patients and methods

### Patients

Patients with stage III and IV operable and inoperable melanoma who were referred to the National Cancer Institute of Naples from July 2006 to December 2008 were enrolled to receive treatment with IFN-α 2b. The study received ethical approval from the 'Comitato Tecnico Scientifico' institutional review board (ref M2/12), and all patients were required to give written informed consent for both the treatment and additional blood samples for serum Treg, TGF-β, IL-10, and autoantibody levels. Before starting treatment all patients were evaluated for disease stage using a whole body computed tomography (CT) scan. Patients were required to meet the following eligibility criteria: stage III melanoma after radical surgery or stage III/IV patients who were unsuitable for primary radical surgery but who may benefit from neoadjuvant IFN-α 2b treatment; ECOG performance status of 0 to 1; adequate hematologic function (whole blood count > 3 × 10^9^/L, neutrophils > 1.5 × 10^9^/L, and platelets > 100 × 10^9^/L); adequate renal function (serum creatinine ≤ 1 × upper limit of normal range [ULN]) and adequate liver function (serum bilirubin < 1.5 × ULN and aspartate transaminase/alanine transaminase ≤ 2.5 × ULN); and adequate cardiac function. Exclusion criteria were: brain metastases (including excised) and disseminated metastatic disease; a history of cardiac disease (e.g., angina, arrhythmia, cardiomiopathy, acute coronary syndrome, and myocardial infarction); uncontrolled diabetes mellitus; thyroid function disorders; a history of psychiatric illness and depression; and a history of autoimmune disease.

### Control groups

The peripheral blood samples of healthy subjects who visited the Transfusion Medicine Unit of the National Cancer Institute of Naples were used as controls for the evaluation of basal Treg levels following assessment of samples for infections and other diseases.

In addition, the peripheral blood samples of patients with stage I to IV melanoma who did not receive treatment with IFN-α 2b were evaluated for basal Treg levels. These patients were also referred to the National Cancer Institute of Naples from July 2006 to December 2008.

### IFN-α 2b treatment

During the first week of treatment, all patients were hospitalized to evaluate tolerability. Providing no severe toxicity was observed, patients were referred to daily outpatient treatment for the remaining 3 weeks. Prior to administration of the first dose it was essential that all blood examinations, electrocardiography, and cardiac ultrasound were normal. IFN-α 2b was administered iv at a dose of 20 MU/m^2 ^each day from day 1 to day 5 for 4 consecutive weeks. Maintenance sc IFN as used in the original E1684 HDI regimen was not included in the therapy planned in this trial [10,14]. A dose reduction of 25% to 30% was permitted if grade 2 to 3 toxicities were observed, and treatment discontinuation was allowed for any grade 4 toxicity or for patient refusal of treatment.

### Follow-up

All patients were followed up between July 2006 and November 2009 to assess DFS (early vs. late recurrence) and survival (alive vs. deceased patients) according to specific National Cancer Institute of Naples internal guidelines. Every 3 months the following assessments were performed for all patients: clinical examination, a regional lymph node ultrasound scan, and an abdominal ultrasound scan. A CT scan was performed every 6 months for patients with stage IV melanoma and every 12 months for patients with stage III melanoma. Magnetic resonance imaging (MRI), positron emission tomography (PET), and bone scintigraphy scans were performed if clinically suspicious signs emerged from the other examinations. Patients were defined as having better prognosis if the disease did not progress during the time of the study. Patients whose disease progressed during the time of the study were defined as having poor prognosis.

### Blood sampling

Whole peripheral blood was collected into EDTA KE/2.7 mL tubes (S-Monovette^®^; Sarstedt, Nümbrecht, Germany) for isolation of peripheral blood mononuclear cells (PBMCs) at days 0, 8, 15, 22, and 29 during IFN-α 2b therapy. An additional 7 mL of peripheral blood was collected in Serum Gel S/7.5 mL plus tubes (S-Monovette^®^; Sarstedt) at the same time as PBMC isolation for assessment of autoantibodies, TGF-β, and IL-10. The serum samples were immediately processed, stored upright for 10 min, then centrifuged at 4°C in a horizontal rotor at 3000 rpm for 10 min. The samples were frozen, stored at -80°C, thawed, and tested simultaneously. Thawed aliquots were used only once.

### Assessment of Treg levels

The Treg levels (%) were measured utilizing a flow cytometry assay for whole lymphocytes in CD4^+ ^cells. The cells were stained with combinations of the following antibodies: anti-CD25-phycoerythrin (PE)–cyanin (Cy) 5.5, anti-CD4-peridinin chlorophyll protein, anti-CD8-allophycocyanin (APC), anti-CD3-APC-Cy7, and isotype controls (BD Biosciences; San Jose, CA, USA). The test tubes were then incubated in the dark for 30 min and then washed with phosphate buffered saline. For intracellular staining of Foxp3-phycoerythrin, anticoagulated whole blood samples were fixed and permeabilized with the use of Foxp3 Staining Buffer Set (eBioscience; San Diego, CA, USA) according to the manufacturer's instructions. The PE-conjugated antibody clone used against Foxp3 was PCH101.

Data acquisition and analysis were performed using the FACSCanto™ II flow cytometry system and FACSdiva™ software (BD Biosciences; San Jose, CA, USA) with a standard 6-color filter configuration. Lymphocytes were gated via their forward and side scatter properties, and T cells were identified based on their expression of CD4 and CD3.

To discriminate between CD25^high ^Treg and CD25^low ^activated effector-memory T cells, we used CD25 expression on CD8^+ ^cells as an internal control. Only CD4^+ ^cells expressing CD25 with higher intensities than the CD8^+ ^cells were included in the gate for CD25^high ^cells. The gate for CD25^low ^cells was set to include cells expressing CD25 at levels above those of the isotype control and unstained cells, but at lower expression levels than the CD25^high ^cells.

This was a phenotypical evaluation of Treg levels, therefore no functional assays or suppression assays were performed to assess Treg levels.

### TGF-β and IL-10 ELISA

Quantitative sandwich enzyme-linked immunosorbent assay (ELISA) was used to measure concentrations of serum TGF-β1 and IL-10, by means of a commercially available kit from Bender MedSystems (Vienna, Austria), according to the manufacturer's instructions.

### Autoantibody detection

Semi-quantitative detection of serum autoantibodies was carried out by assessing antinuclear antibody (anti-ANA), anti-cardiolipin antibodies (anti-ACA immunoglobulin [Ig]; QUANTA Lite™ ELISA kit; INOVA Diagnostics, Inc., San Diego, CA, USA), anti-double stranded DNA (anti-dsDNA), and anti-thyroglobulin (anti-HTG; Roche Diagnostics GmbH, Mannheim, Germany). Each patient sample was diluted 1:101 with horseradish peroxidase (HRP) sample diluent and run in duplicate; the method required prediluted ELISA calibrator, and ELISA negative and positive controls. A 100 μL aliquot of each sample was added to the microwell plate and incubated for 30 min at room temperature. After 3 washes with HRP, the wash buffer was diluted 1:40, then 100 μL of the HRP-IgG conjugate was added to each well, and the plate incubated for 30 min at room temperature. After 3 washes, 100 μL of 3,3′,5,5′-tetramethylbenzidine (TMB) chromogen was added to each well, and the wells incubated in the dark for 30 min at room temperature followed by the addition of 100 μL of HRP stop solution. The absorbance of each well was measured at reference wavelengths of 450 nm and 620 nm.

### Efficacy outcomes

The primary outcome measure in the neoadjuvant setting was change in tumor size and status, and the consideration of operability from inoperable to operable following therapy (neoadjuvant setting) or DFS status (standard postoperative adjuvant setting). Response evaluation criteria in solid tumors (RECIST) were used to evaluate tumor response. RFS in the entire and adjuvant/neoadjuvant patient populations was also evaluated in this study.

### Safety

The National Cancer Institute-Common Terminology Criteria for Adverse Events (NCI-CTCAE) version 3.0 was used to assess toxicity.

### Statistical methods

All analyses were performed using the Statistical Package for Social Sciences for Windows, following recommended procedures (SPSS^® ^for Windows Version 12.0; SPSS, Inc., Chicago, IL, USA).

Levels of Treg (%), TGF-β (ng/mL), IL-10 (pg/mL), and autoantibodies (U/L or IU/mL) were scrutinized using descriptive procedures. The distributions of these values were compared by time (weeks) from serum drawing, and basal values were also compared by disease stage (I to IV), treatment (adjuvant vs. neoadjuvant), prognosis (early recurrence vs. no recurrence), and disease status at follow-up (alive vs. deceased). Box plots were used to show distributions in different patient subgroups. In each box plot, median values are represented by the horizontal line inside the boxes, the upper and lower boundaries of the boxes indicate first and third quartiles of the distribution, whiskers represent mild outliers (i.e., values lying within 1.5 box lengths from either end of the box), open dots are outliers (i.e., values lying between 1.5 and 3 box lengths from either end of the box), and asterisks represent extreme outliers (i.e., values lying more than 3 box lengths from either end of the box). The Mann-Whitney test was used to compare median values of Tregs between subgroups of patients at baseline according to treatment type, prognosis, stage, status at follow-up, between treated patients versus healthy donors, and both untreated and treated patients versus healthy donors. In each case, significance was established as *P *< 0.05. Mean percentage Treg levels were also calculated to verify the weekly percentage variation. RFS in the entire and adjuvant patient populations was estimated using the Kaplan-Meier method.

## Results

### Patient characteristics

Patient characteristics are shown in Table 1 for the 22 patients treated with IFN-α 2b, the 22 patients not treated with IFN-α 2b, and the 20 healthy subjects. Of the 22 treated patients, 17 (77%) received postoperative adjuvant IFN-α 2b therapy and 5 (23%) received preoperative neoadjuvant IFN-α 2b therapy. Two of the stage IV patients had lung metastases and 1 had distant lymph node metastases. At the time of analysis, the 22 patients with melanoma who did not receive treatment with IFN-α 2b were at the following stages of disease: stage I (n = 4); stage II (n = 2); stage III (n = 6, refused treatment); and stage IV (n = 10).

**Table 1:** Subject characteristics

	IFN-α 2b-treated patients with melanoma	Untreated patients with melanoma	Healthy subjects	IFN-α 2b therapy, n (%)	
				
				Adjuvant	Neoadjuvant
Subjects, n	22	22	20		
Mean age, years	43.0	43.5	44.8		
Sex, n (%)					
Male	12 (55)	10	13		
Female	10 (45)	12	7		
Melanoma stage, n (%)					
I	N/A	4 (18.3)	N/A	N/A	N/A
II	N/A	2 (9.2)	N/A	N/A	N/A
IIIA	12 (54.5)	2* (9.2)	N/A	12 (54.5)	0
IIIC	3 (13.7)	4* (18.3)	N/A	1 (4.5)	2 (9.1)
IV	7 (31.8)	10 (45)	N/A	4 (18.2)	3 (13.7)

N/A, not applicable; IFN-α 2b, interferon-α 2b.

*Refused treatment.

**Figure 1:**
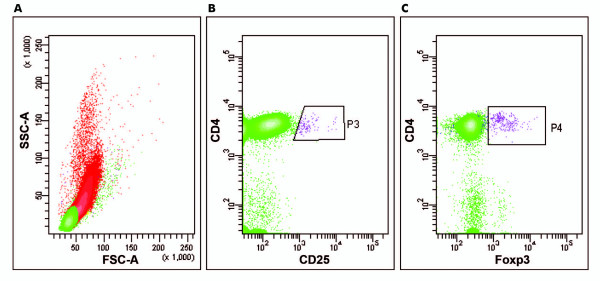
**Flow cytometry: percentage of CD4^+^CD25^+HF^Foxp3^+ ^cells in interferon-α 2b-treated patients with melanoma**. Flow cytometric gating strategy to identify Treg cells. (**A**) Dot plot of forward scatter (FSC) versus side scatter (SSC) for all events: all peripheral blood cell populations are shown in red; the population of lymphocytes is shown in green. (**B**) Lymphocytes (green) were analyzed on the basis of surface markers CD4 and CD25; the P3 gate identifies the percentage of CD4^+^CD25^+HF ^cells. (**C**) The P4 gate identifies the percentage of Foxp3^+^CD4^+^CD25^+HF ^cells; this represents the region used to calculate the final percentage of Treg cells in CD4^+ ^lymphocytes.

The Treg levels (%) were established using flow cytometric analysis (on days 0, 8, 15, 22, and 29) of the peripheral blood of each patient with melanoma treated with IFN-α 2b. An example of the gates for CD4^+^CD25^+HF ^and CD4^+^CD25^+HF^Foxp3^+ ^are shown in Figure 1, which enabled the evaluation of the percentage of CD4^+^CD25^+HF^Foxp3^+ ^cells. Region P4 on Figure 1C represents the Foxp3^+^CD4^+^CD25^+ ^cells used to calculate the percentage of Treg cells in CD4^+ ^lymphocytes.

**Figure 2:**
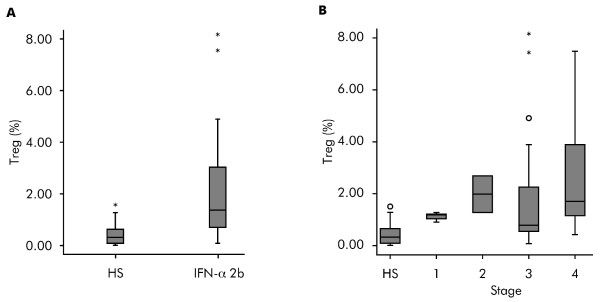
**Comparison of regulatory T cell levels at baseline in patients with melanoma and healthy subjects**. (**A**) Box plot showing baseline regulatory T cell (Treg) levels in 22 patients with melanoma prior to interferon-α 2b (IFN-α 2b) treatment compared with 20 healthy subjects (HS) (*P = *0.001). (**B**) Box plot showing Treg levels by disease stage in 20 healthy subjects and 44 patients with melanoma (*P <*0.01 for increased Tregs in all melanoma patients vs. healthy subjects); *P *= not significant for Treg increase by disease stage. Horizontal lines inside the boxes = median values; upper and lower boundaries of the boxes = first and third quartiles of the distribution; whiskers = mild outliers; open dots = outliers; and asterisks = extreme outliers.

As shown in Figure 2A, higher basal Treg values were observed in the 22 patients with melanoma before treatment with IFN-α 2b compared with the 20 untreated healthy subjects (*P = *0.001). Figure 2B shows the Treg basal level distribution in the 20 healthy donors and by stage in the 44 patients with melanoma (22 patients treated in this study and the other 22 patients who were referred to our institution during the study); these data again suggest that Treg values are higher in patients with melanoma (*P <*0.01 for increased Tregs in all melanoma patients vs healthy subjects). There was a trend for an increase in Treg levels by increase in stage of disease, but this was not statistically significant.

**Figure 3:**
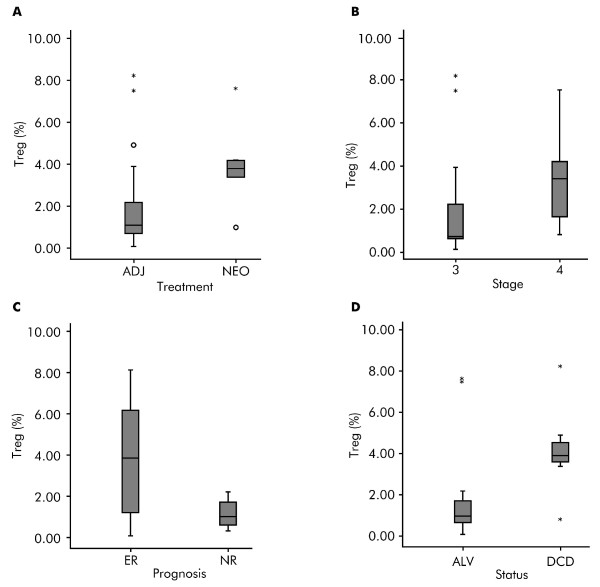
**Subgroup comparisons: regulatory T cell levels at baseline in patients with melanoma before treatment with interferon-α 2b**. Box plot subgroup comparisons of regulatory T cell (Treg) levels (%) at baseline in 22 patients with melanoma before treatment with interferon-α 2b (IFN-α 2b). (**A**) Adjuvant (ADJ) versus neoadjuvant (NEO) IFN-α 2b (*P = *not significant); (**B**) stage III versus stage IV (*P = *0.082); (**C**) early recurrence (ER) versus no recurrence (NR) (*P = *0.017); (**D**) surviving (ALV) versus deceased (DCD) (*P = *0.021). Horizontal lines inside the boxes = median values; upper and lower boundaries of the boxes = first and third quartiles of the distribution; whiskers = mild outliers; open dots = outliers; and asterisks = extreme outliers.

Subgroup comparisons for basal Treg levels before treatment are shown in Figures 3A-D. Although not statistically significant, patients scheduled to receive IFN-α 2b in the postoperative adjuvant setting had lower basal Treg levels than those in the preoperative neoadjuvant group, as illustrated in Figure 3A. At baseline, patients with stage III melanoma had lower Treg levels than stage IV patients (*P *= 0.082; Figure 3B). Figure 3C illustrates that patients with early recurrence exhibited significantly greater basal Treg values than those with no recurrence (*P *= 0.017). Significantly lower basal Treg values were measured in surviving patients at the time of analysis compared with patients that later deceased (*P = *0.021; Figure 3D).

### Effects of IFN-α 2b on Tregs

**Figure 4:**
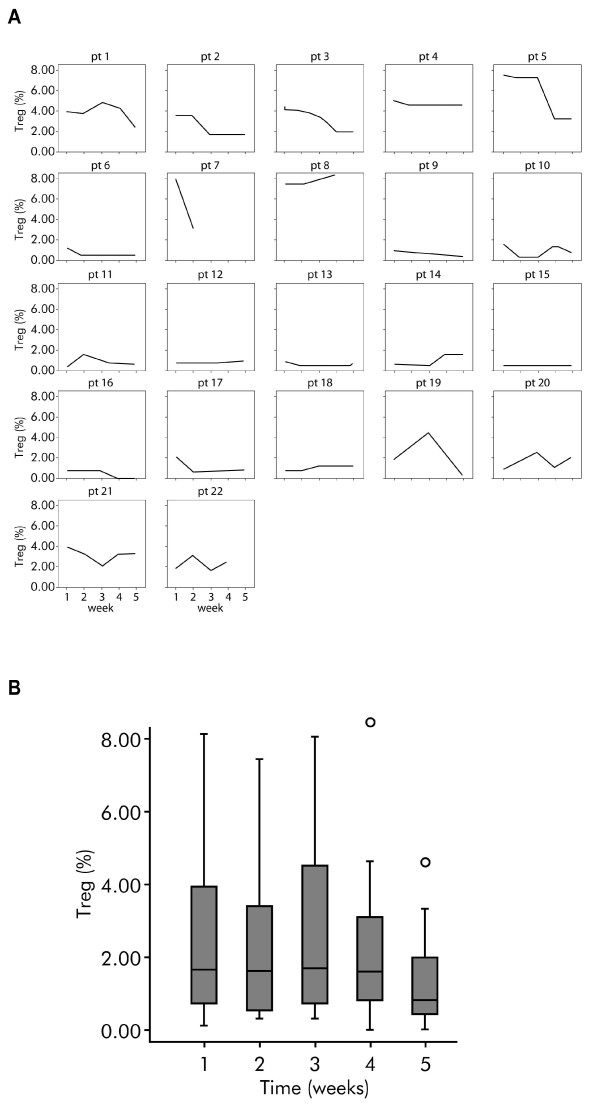
**Regulatory T cell levels by week in interferon-α 2b-treated patients with melanoma**. (**A**) Individual regulatory T cell (Treg) levels by week in the 22 patients with melanoma treated with interferon-α 2b. (**B**)Box plot showing circulating regulatory T cell (Treg) levels by week in the 22 patients with melanoma treated with interferon-α 2b (*P = *not significant). Horizontal lines inside the boxes = median values; upper and lower boundaries of the boxes = first and third quartiles of the distribution; whiskers = mild outliers; and open dots = outliers.

The Treg levels by week in patients treated with IFN-α 2b are shown in Figures 4A (individual patients) and B (all patients). For individual patients (Figure 4A), 14 of 22 (63.6%) patients showed a decrease in Treg cells in peripheral blood during treatment with IFN-α 2b, in 1 patient there was no change (#15), while in all the other patients an increase in Treg was observed. Overall, a gradual decrease in Treg values was observed over time, although this was not statistically significant (Figure 4B). The mean percentage of Tregs was 2.7% at day 0 and 1.4% at day 29. The average reduction was 1.4%, representing a 50% reduction in the average Treg levels. Statistical analysis showed an average decrease of 0.29% per week of treatment (mean data not shown).

Following treatment with IFN-α 2b, no statistically significant differences were observed in the patient subgroups analyzed (adjuvant vs neoadjuvant IFN-α 2b, stage III vs stage IV, early recurrence vs no recurrence and surviving vs deceased) from baseline to the end of the study (data not shown).

### Effect of IFN-α 2b on TGF-β, IL-10, and autoantibody levels

**Figure 5:**
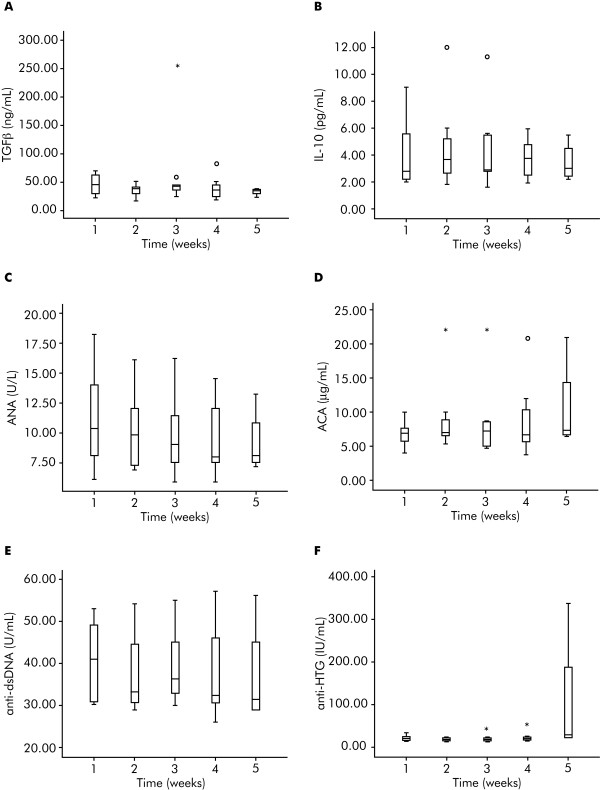
**Determination of transforming growth factor-β, interleukin-10 and autoantibody in interferon-α 2b-treated patients with melanoma**. (**A**) Transforming growth factor-β (TGF-β), (**B**) interleukin (IL)-10, and serum autoantibodies: (**C**) antinuclear antibody (ANA), (**D**) anti-cardiolipin (ACA), (**E**) anti-double stranded DNA (anti-dsDNA), and (**F**) anti-thyroglobulin (anti-HTG) levels by week in 14/22 patients with melanoma treated with interferon-α 2b. Horizontal lines inside the boxes = median values; upper and lower boundaries of the boxes = first and third quartiles of the distribution; whiskers = mild outliers; open dots = outliers; and asterisks = extreme outliers.

In addition to Tregs, the levels of TGF-β IL-10, and autoantibodies (ANA, ACA, anti-dsDNA, and anti-HTG) were measured in serum samples collected from 14 of the 22 patients with melanoma undergoing IFN-α 2b treatment in this study. No significant effects were observed (Figure 5).

### Efficacy

**Figure 6:**
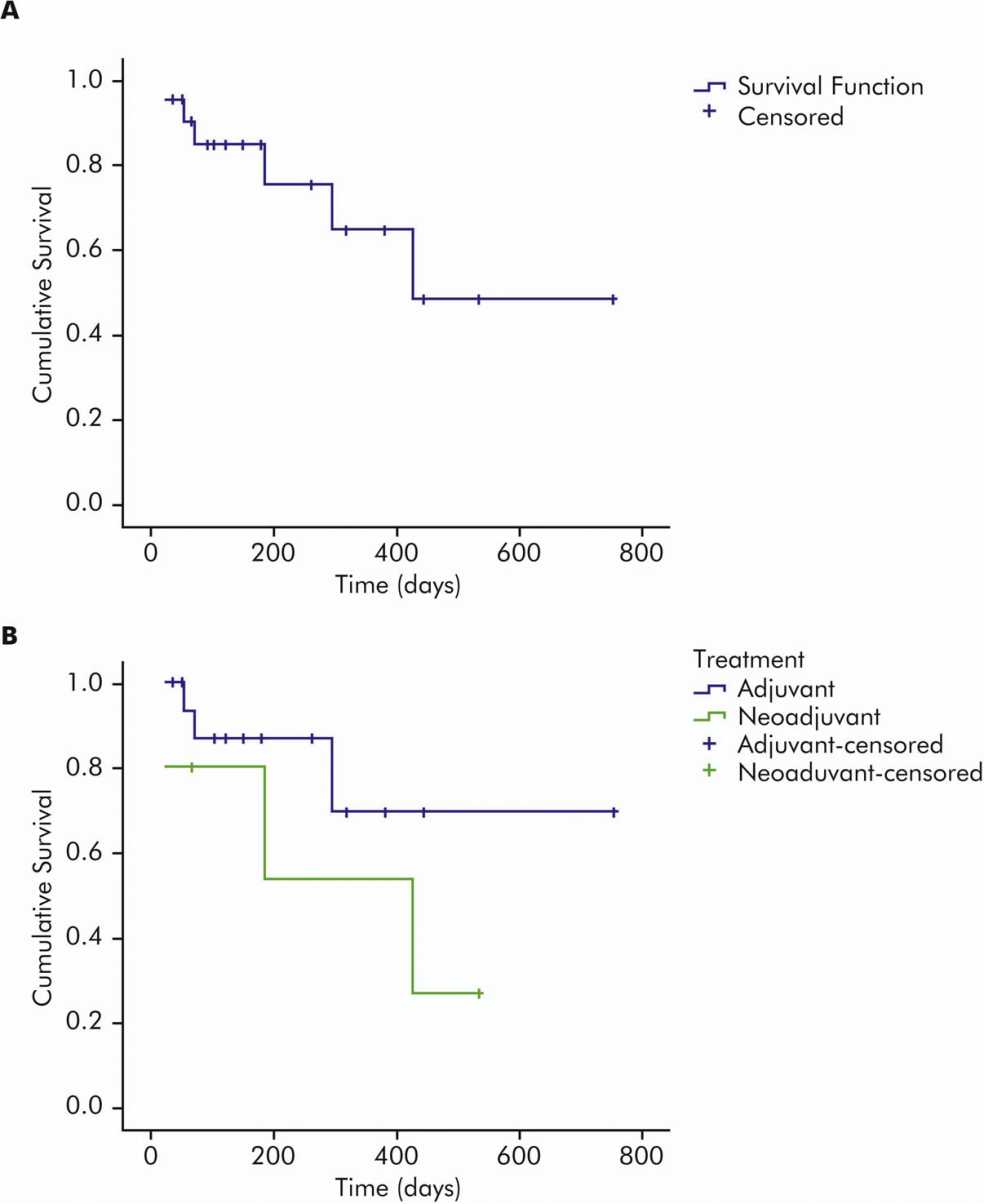
**Kaplan-Meier relapse-free survival curves**. Kaplein-Meier relapse-free survival curves for (**A**) all 22 interferon-α 2b-treated patients with melanoma and (**B**) patients receiving neoadjuvant versus adjuvant therapy.

Kaplan-Meier curves for RFS in all 22 IFN-α 2b-treated patients with melanoma and in the adjuvant/neoadjuvant populations are shown in Figures 6A and 6B, respectively. The mean RFS estimates were 16.4 months (95% CI: 11.0-21.7), 19.4 months (95% CI: 13.6-25.1) and 10.3 months (95% CI: 4.2-16.4) for the entire population, the adjuvant population and the neoadjuvant population, respectively (median estimates not calculable).

Of the 5 patients who received neoadjuvant IFN-α 2b therapy, 1 underwent radical surgery and had a RFS of 17 months and 1 had a complete response that was maintained at a follow-up of 19 months. The other 3 patients progressed and then received chemotherapy. Two patients received 6 cycles of dacarbazine (1000 mg/m^2 ^every 3 weeks), with progressive disease and had died at the time of analysis; the other patient received a combination of cisplatin (75 mg/m^2 ^on day 1 of a 28-day cycle) and temozolomide (75 mg/m^2 ^per day from day 2 to day 22 of a 28-day cycle) [31], and after 3 cycles had partial response in liver metastases. This patient subsequently underwent surgery for liver metastases and was disease-free for 12 months. At the time of analysis this patient was still alive and was receiving a second line of chemotherapy with fotemustine following the reappearance of liver and lung metastases.

Of the 17 patients treated with adjuvant IFN-α 2b, 4 patients had very rapid progressive disease and died following chemotherapy. Nine of 13 patients with disease recurrence started chemotherapy (cisplatin and temozolomide as described above) [31]. Four of these patients had progressive disease after 3 cycles and died; the other 5 had stable disease for 6 months and at the time of analysis were still alive.

### Safety

Adverse events are summarized in Table 2. Hepatic toxicity was the most frequent side effect, resulting in a 25% dose reduction in 10 patients. In general, nausea and vomiting were more frequent during the first week of treatment. Although they did not bring about therapy discontinuation, the most frequent hematologic side effects were lymphocytopenia and neutropenia, occurring at grade III in 2 (9%) and 3 (14%) patients, respectively. No grade IV adverse events or autoimmunity side effects were observed in this study.

**Table 2:** All adverse events observed in the 22 interferon-α 2b-treated patients with melanoma

	Grade, n (%)			
	
Adverse events*	I	II	III	IV
Hematologic				
Neutropenia	7 (31)	5 (23)	3 (14)	0
Lymphocytopenia	10 (45)	5 (23)	2 (9)	0
Liver function				
AST	15 (68)	13 (59)	10 (45)	0
ALT	20 (91)	15 (68)	12 (54)	0
Nonhematologic				
Fatigue	17 (77)	6 (27)	2 (9)	0
Nausea	8 (36)	7 (32)	2 (9)	0
Vomiting	5 (22)	4 (18)	2 (9)	0
Anorexia	9 (40)	3 (14)	0	0
Diarrhea	3 (14)	0	0	0
Flu-like syndrome^†^	17 (77)	7 (31)	0	0
Autoimmunity	0	0	0	0

AST, aspartate transaminase; ALT, alanine transaminase.

*Assessed using the National Cancer Institute-Common Terminology Criteria for Adverse Events version 3.0.

^†^Flu-like syndrome includes headache, fever, myalgia, and chills.

## Discussion

This study aimed to provide an insight into the mechanism of action of IFN-α 2b in the adjuvant treatment of melanoma. A link between an immunologic response, measured by Treg cell numbers, and antitumor activity was investigated.

We evaluated only the induction phase of the FDA-approved HDI regimen, which is now prospectively being tested in the ECOG trial E1697 in comparison with observation. The dose and duration of therapy as selected was based upon the literature and meta-analyses, which now raise the question as to whether the iv induction regimen of the E1684 HDI regimen is the active component of the 1-year regimen [10,14]. Following recent publications relating to the reduction of dose and duration of IFN-α 2b [11], there is an ongoing debate [2,12] in the melanoma treatment community regarding the optimum dosing schedule and duration of treatment for IFN-α 2b in the adjuvant treatment of melanoma.

The data presented support the earlier results of Cesana *et al*. [28] and Tatsugami *et al*. [29] suggesting that both patients with melanoma and RCC have increased basal levels of Treg that may be relevant to their disease and its progression. At baseline, higher Treg levels were observed in patients with melanoma compared with healthy subjects and the levels were significantly greater in patients with more advanced disease. Subgroup comparisons showed that substantial variability in Treg levels was observed according to disease stage, outcome (recurrence/no recurrence, alive/deceased), and type of treatment received (adjuvant/neoadjuvant). Subgroup analyses according to prognosis and disease outcome suggest that lower Treg values are associated with better prognosis and greater chance of survival in patients with melanoma. Statistically significant correlations were measured for higher baseline Treg levels for stage III versus stage IV disease (*P = *0.082), early recurrence versus no recurrence (*P *= 0.017) and deceased versus surviving patients (*P = *0.021). Although a clear trend for a reduction in Treg levels with HDI induction therapy in patients with melanoma was observed, statistical significance was not reached. This may be due, in part, to the small size of this study. In addition, it should be noted that because this was a phenotypical evaluation of Treg levels, no functional assays or Treg suppression assays were performed for assessment of Treg levels. Another possible caveat of the method used in this study is that surface CD25 and intracellular Foxp3 expressions are not strictly specific of Treg cells in humans, as both these markers are upregulated after activation in a significant proportion of CD4^+ ^non-regulatory T cells.

Several studies have shown that the appearance of autoantibodies and clinical manifestations of autoimmunity were associated with significant improvements in RFS and OS in patients with melanoma treated with IFN-α 2b [16-18]. These data were not substantiated in our study where no statistically significant effect of IFN-α 2b on serum TGF-β, IL-10, and serum autoantibodies was observed among the patients with melanoma; again perhaps because of the small size of the sample. These preliminary data have suggested that the evaluated immunoregulatory proteins and autoantibodies are not modulated by IFN-α 2b within the short interval studied here.

Although not the main focus of this pilot study, efficacy of IFN-α 2b was assessed. In the 5 patients receiving neoadjuvant IFN-α 2b, 1 complete response was observed that was maintained at a follow-up of 19 months. Another patient had a RFS of 17 months after neoadjuvant therapy and radical surgery. Of the 17 patients receiving postoperative adjuvant IFN-α 2b, 13 developed disease recurrence. The toxicity profile of IFN-α 2b was as expected based on previous clinical experience [13]. A 25% dose reduction was required in 10 of 22 patients due to hepatic toxicity. No evidence of autoimmunity was observed.

## Conclusions

The main limitation of this study is its small sample size. Our preliminary data suggest that Treg levels shift during treatment with IFN-α 2b, although a direct effect was not shown. There was no correlation of Treg levels with either objective response or survival, and no conclusion regarding the role of Tregs in terms of response to treatment or as a prognostic marker of outcome can be inferred at this stage. Further data are awaited in order to examine whether affecting Treg levels with IFN-α 2b treatment may indeed contribute to the antitumor response. A better understanding of the mechanism of action of IFN-α 2b may facilitate the development of treatment strategies to increase efficacy and reduce toxicity, ultimately leading to a better standard of care for patients with melanoma.

## Competing interests

PAA participated in an advisory board for Bristol-Myers Squibb and has received honoraria from Schering-Plough and Genta. JMK receives research funding to the University of Pittsburgh from BMS, Pfizer, Lilly, and Intrexon, and is on the Speaker Bureau of Schering-Plough. The other authors have no competing interests to declare.

## Authors' contributions

PAA and FMM were responsible for the conception and design of the study. PAA, MN, ES, GG, AD, MCa, CC, RC, GB, MCe, GC, and NM were responsible for provision of study materials or patients. MN, GG, and MCa collected and assembled data for the Treg assay. RC and GC were responsible for the TGF-β and IL-10 assays. MCe and GB were involved with the autoantibody assay. VDR was responsible for radiologic evaluation for neoadjuvant patients. Patient follow-up was performed by ES, AD, CC, and NM. EC, PAA, GP, JMK, and FMM contributed to data analysis and interpretation. All authors were involved in manuscript writing and provided final approval of the manuscript.
